# Supplementary material for: Research on China-EU equipment manufacturing Trade dependence in intra-industry specialization view
Source: PLoS One. 2022 Nov 28;17(11):e0278119. doi: 10.1371/journal.pone.0278119 (PMC9704630; doi:10.1371/journal.pone.0278119)
Supplement: Attachment — Submitted filename: Response to Reviewers.docx [file pone.0278119.s001.docx]

Dear Editor and Reviewer：

Thank you for giving us the opportunity to revise our paper. We have made a lot of efforts to improve the paper according to your suggestions during this period.Here below is our description on revision according to the comments.

**Part A （To Academic Editor）**

1. We think it is wrong to describe our research from the perspective of value chain. Value chain should mean that different segments of a product are produced by different countries. What we study is the division of labor within the industry, which is an unequal concept. Therefore, we have revised the part of the value chain.

2. In the conclusion part, we added data explanation to make the conclusion less abrupt.

**Part A （To Reviewer）**

1. The introduction has been revised. We are no longer limited to the perspective of China, but focus on the study of the characteristics of trade relations. The purpose and significance of the study have also been supplemented

2. In sorting out the existing research, the research method of trade dependence is changed to the influencing factors of trade dependence, and the quality of the cited literature is also paid more attention to.

3. Added the definition of Herfindahl Hirschman index and its difference from other indexes.

4. The conclusion part has been modified to give more explanation and discussion. At the same time, it complements the direction of future research.

Thank academic editor and reviewer for your suggestions. We have learned a lot from them, which has provided a lot of help for our research. Although the existing modifications can not be perfect, we have gained a lot in the process of in-depth research.

Sincerely Yours,

Lingxiang Jian, Tiantian Ding, Wanyun Ma
